# Supplementary material for: Kinetically-controlled intermediate-direct-pinning for homogeneous energy landscapes in quasi-two-dimensional perovskites for efficient and narrow blue emission
Source: Nat Commun. 2025 Oct 30;16:9590. doi: 10.1038/s41467-025-62863-y (PMC12575853; doi:10.1038/s41467-025-62863-y)
Supplement: Supplementary file 1 — Supplementary Information [file 41467_2025_62863_MOESM1_ESM.docx]

**Kinetically-controlled intermediate-direct-pinning for homogeneous energy landscapes in quasi-two-dimensional perovskites for efficient and narrow blue emission**

Joo Sung Kim^1,2,3,†^, Hyeon-Dong Lee^1,†^, Jeong Hyun Jung^1,†^, Taehee Kim^4,†^, Seung-Je Woo^1^, Hyung Joong Yoon^5^, Jaehyun Moon^6^, Chan-mo Kang^6^, Seung-Eui Chang^1^, Dong-Hyeok Kim^1^, Sungjin Kim^1^, Hoichang Yang^7^, Dongho Kim^4*^, and Tae-Woo Lee^1,2,3,8*^

*^1^Department of Materials Science and Engineering, Seoul National University, 1 Gwanak-ro Gwanak-gu, Seoul 08826, Republic of Korea*

*^2^Soft Foundry, Seoul National University, 1 Gwanak-ro, Gwanak-gu, Seoul 08826, Republic of Korea*

*^3^SN DISPLAY Co. Ltd., Building 33, 1 Gwanak-ro, Gwanak-gu, Seoul, Republic of Korea*

*^4^Department of Chemistry, Yonsei University, 50 Yonsei-ro, Seodaemun-gu, Seoul 03722, Republic of Korea*

*^5^Research Center for Materials Analysis, Korea Basic Science Institute (KBSI), 169-148 Gwahak-ro, Yuseong-gu, Daejeon, 34133, Republic of Korea*

*^6^Reality Devices Research Division, Electronics and Telecommunications Research Institute, 218, Gajeong-ro, Yuseong-gu, Daejeon, 34129, Republic of Korea*

*^7^Department of Chemical Engineering, Inha University, 100 Inha-ro, Michuhol-gu, Incheon, 22212, Republic of Korea*

*^8^Interdisciplinary Program in Bioengineering, Institute of Engineering Research, Research Institute of Advanced Materials, Seoul National University, 1 Gwanak-ro, Gwanak-gu, Seoul 08826, Republic of Korea.*

^†^*These Authors contributed equally to this work*

**Authors to whom correspondence should be addressed: E-mail: twlees@snu.ac.kr, dongho@yonsei.ac.kr*

This file contains

**Supplementary Fig. 1 |** Schematic representation of fabrication procedure.

**Supplementary Fig. 2** | *In-situ* monitored crystallization kinetics of perovskite thin films.

**Supplementary Fig. 3 |** Effect of A-IDP process on morphology of quasi-2D perovskite thin films.

**Supplementary Fig. 4** | Effect of A-IDP process on crystal structure of quasi-2D perovskite thin films.

**Supplementary Fig. 5 |** Effect of A-IDP process on nanostructure of quasi-2D perovskite thin films.

**Supplementary Fig. 6** | Chemical analysis on A-IDP process.

**Supplementary Fig. 7** | PL lifetime spectra of pristine and A-IDP films.

**Supplementary Fig. 8** | Solution-state chemical analysis on cation-π charge-transfer complex.

**Supplementary Fig. 9 |** ^1^H 2D DOSY spectra of perovskite precursor solutions.

**Supplementary Fig. 10 |** Effect of MDACl_2_ additives on nanostructure of perovskite thin films.

**Supplementary Fig. 11 |** Effect of MDACl_2_ additives on crystal structure of perovskite thin films.

**Supplementary Fig. 12** | Steady-state PL spectra of pristine perovskite films with different amounts of MDACl_2_.

**Supplementary Fig. 13 |** Effect of CCA on charge carrier dynamics of perovskite thin films.

**Supplementary Fig. 14** | Phase distribution analysis with multiple Gaussian functions.

**Supplementary Fig. 15** | TA dynamics at higher pump fluences.

**Supplementary Fig. 16** | Electrical property of quasi-2D perovskite LEDs.

**Supplementary Fig. 17 |** Conductivity measurements for the lateral devices of quasi-2D perovskite thin films.

**Supplementary Fig. 18 |** Angle-dependent EL intensity of PeLEDs.

**Supplementary Fig. 19 |** Device characteristics of PeLEDs with different amount of MDACl_2_.

**Supplementary Fig. 20 |** Device characteristics of PeLEDs with different amount of CCA.

**Supplementary Fig. 21** | Emission wavelength tunability of A-IDP PeLEDs.

**Supplementary Table 1 |** Summarized electrical and luminance characteristics of PeLEDs.

**Supplementary Table 2 |** Summarized electrical and luminance characteristics of pristine and A-IDP PeLEDs with different MDACl_2_ ratio.

**Supplementary Table 3** | Summary of reported high-efficiency blue PeLEDs (without an outcoupling strategy).


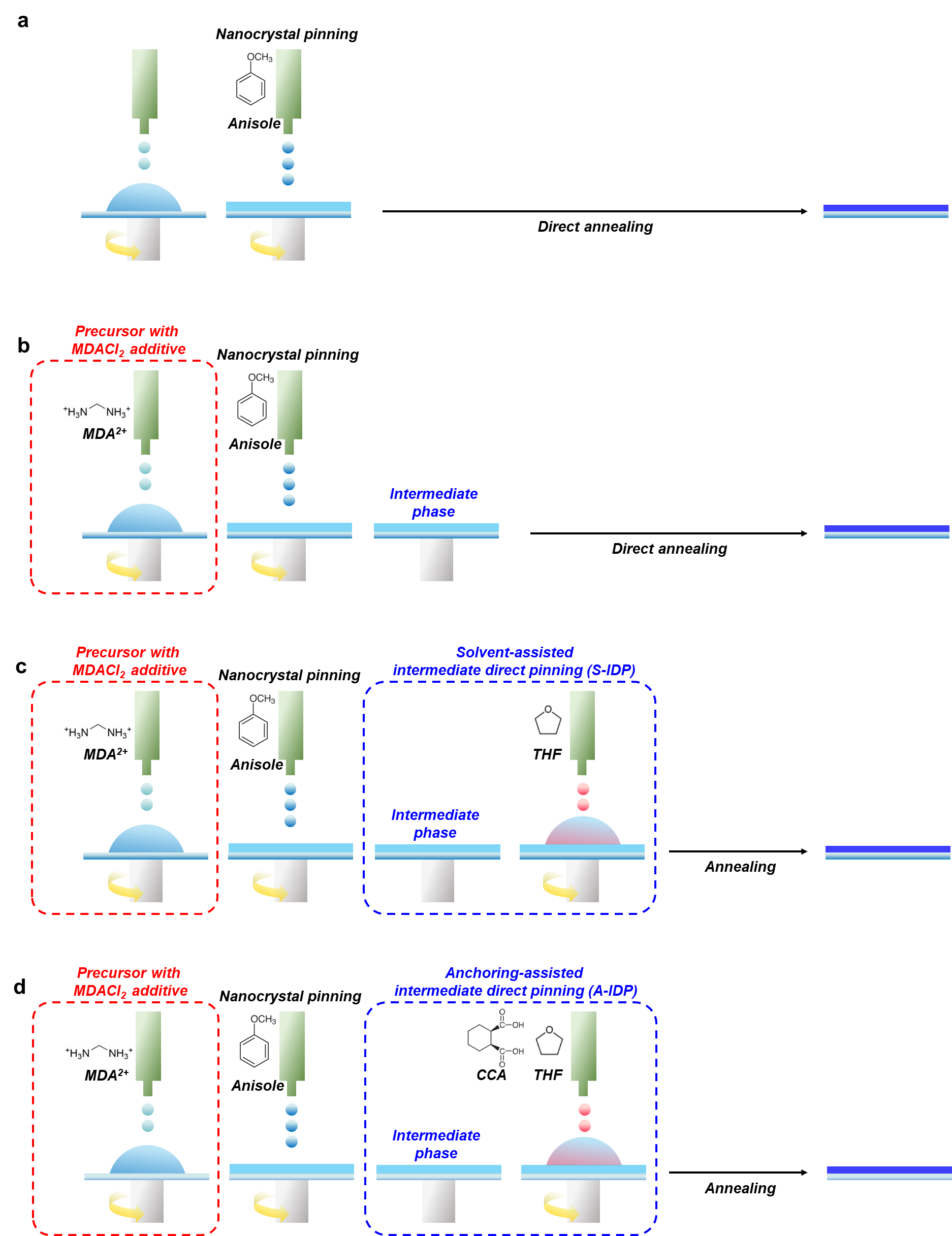


**Supplementary Fig. 1 | Schematic representation of fabrication procedure.
a**, Perovskite film fabrication with one-step precursor-to-crystal conversion. The nanocrystal-pinning process was applied to achieve full-coverage film without pinholes, followed by direct annealing. **b**, Pristine perovskite film fabrication with intermediate stage induced by MDACl_2_ additive into precursor solution. Without an intermediate pinning process, the direct annealing of mobile intermediate phases can undergo diffusion-induced phase broadening toward thermodynamically stable low-*n* phases. **c**, Perovskite film fabrication with intermediate stage and S-IDP process. **d**, Perovskite film fabrication with intermediate stage and A-IDP process.


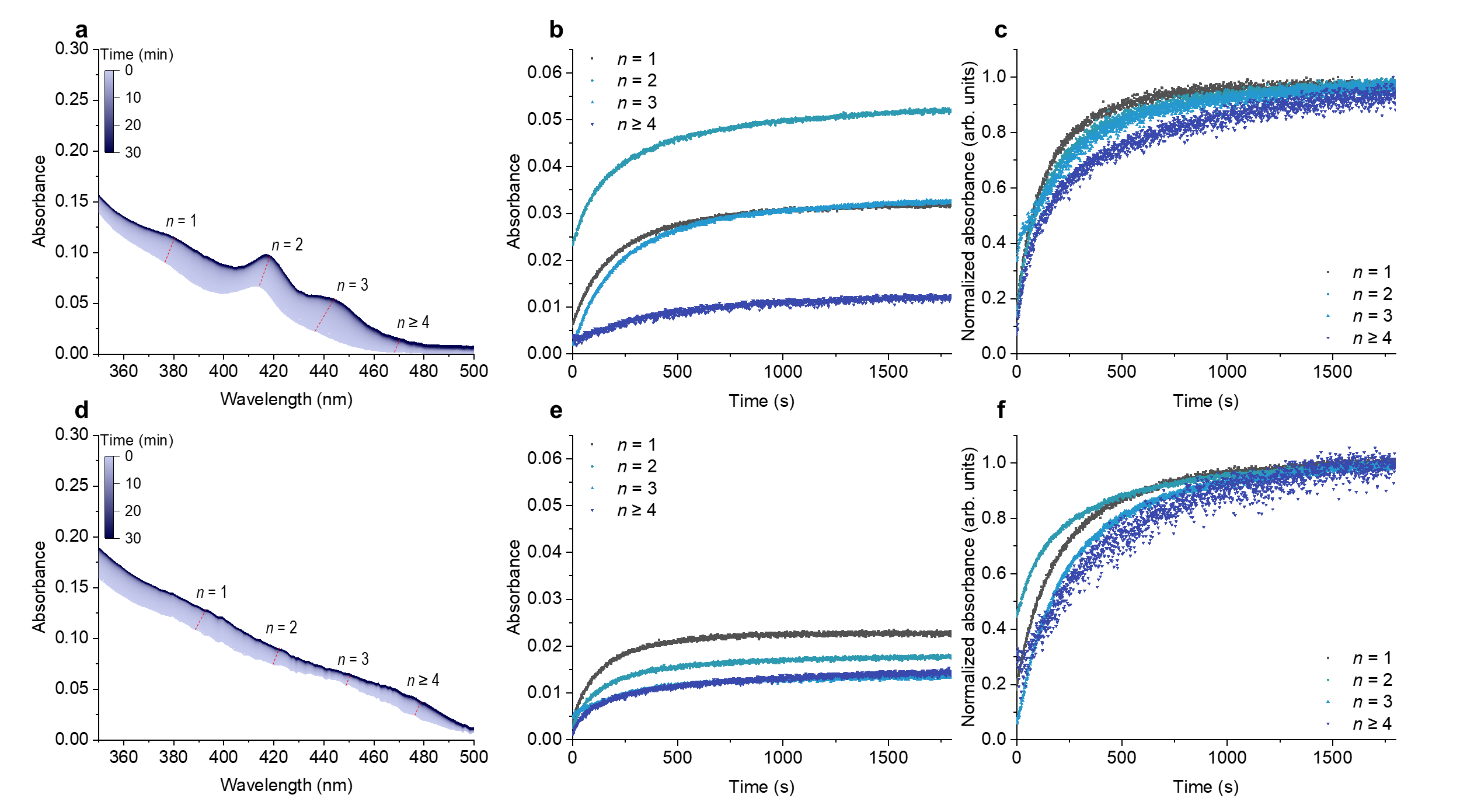


**Supplementary Fig. 2 | *In-situ* monitored crystallization kinetics of perovskite thin films.** *In-situ* monitored UV-vis absorption spectra of pristine (**a**) and A-IDP (**d**) perovskite films during the crystallization process. The absorbance of each *n* phases (*n=*1, 2, 3, ≥4) (**b**, **e**) and normalized absorbance (**c**, **f**) of pristine and A-IDP perovskite films during the crystallization process. UV-Vis spectra of perovskite films were monitored right after the end of spin coating. The absorption edge of each *n*=1, 2, 3, and ≥4 phases are marked with line and used to estimate the quantitative distribution of each phase.


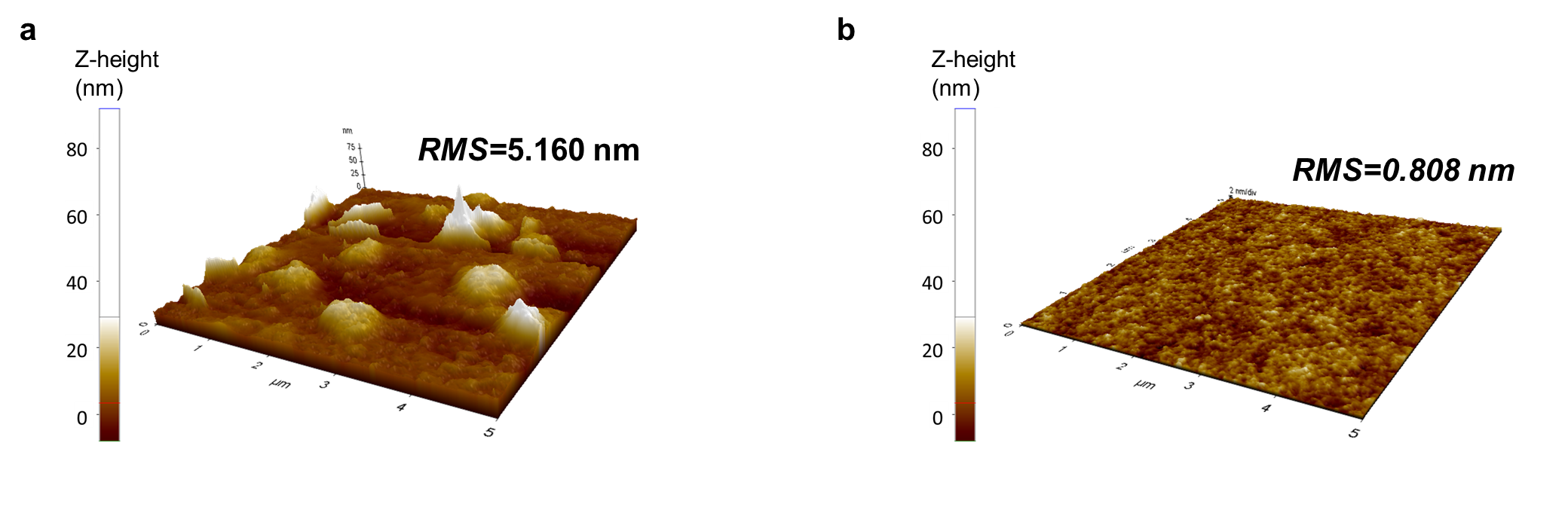


**Supplementary Fig. 3 | Effect of A-IDP process on morphology of quasi-2D perovskite thin films. a-b**, AFM image of perovskite thin films before (**a**) and after (**b**) A-IDP process.


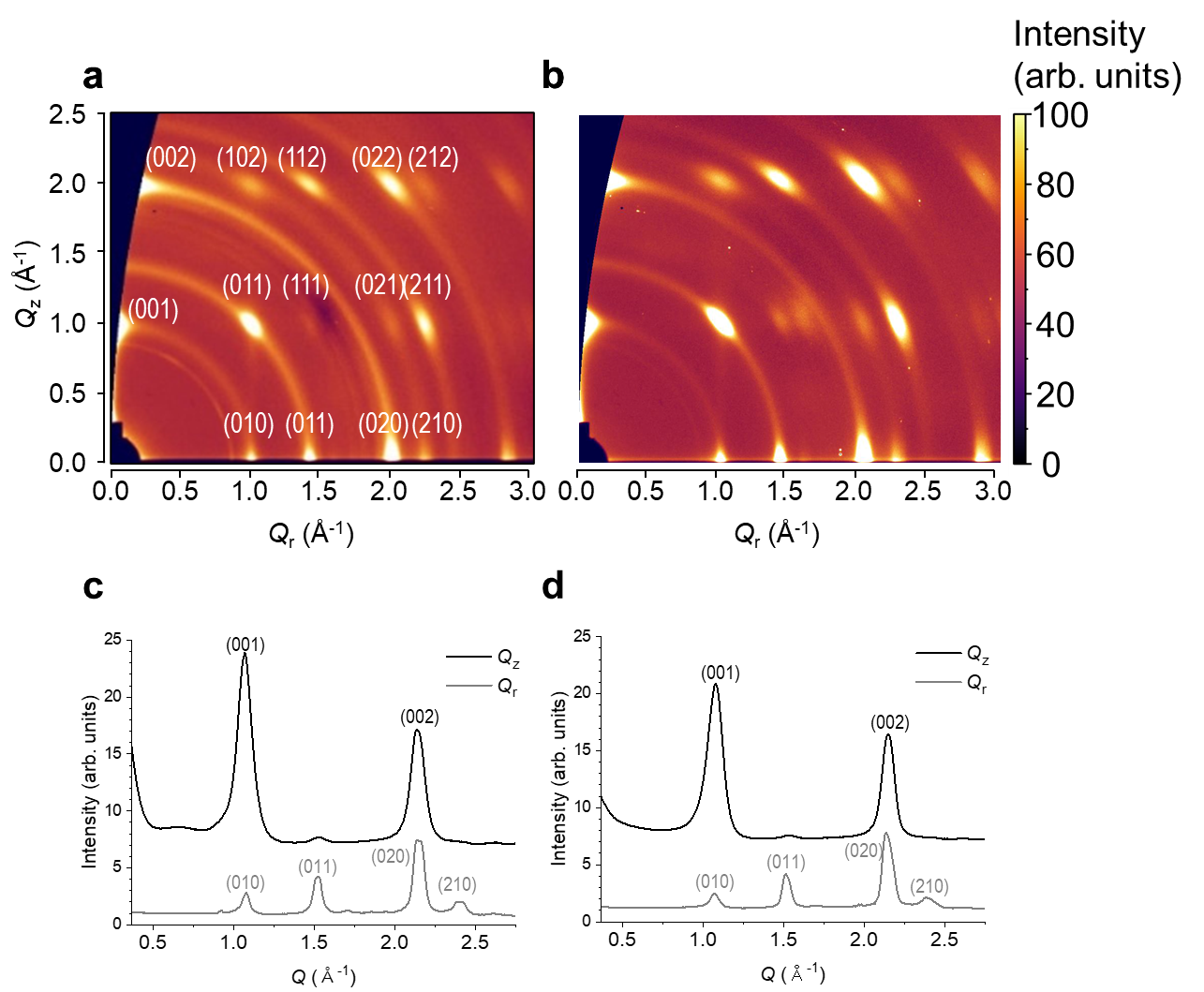


**Supplementary Fig. 4 | Effect of A-IDP process on crystal structure of quasi-2D perovskite thin films.** GIWAXS and 1D XRD profile of perovskite thin film with CCA-THF solution exposure time of 10 s (**a**, **c**) and 20 s (**b**, **d**).


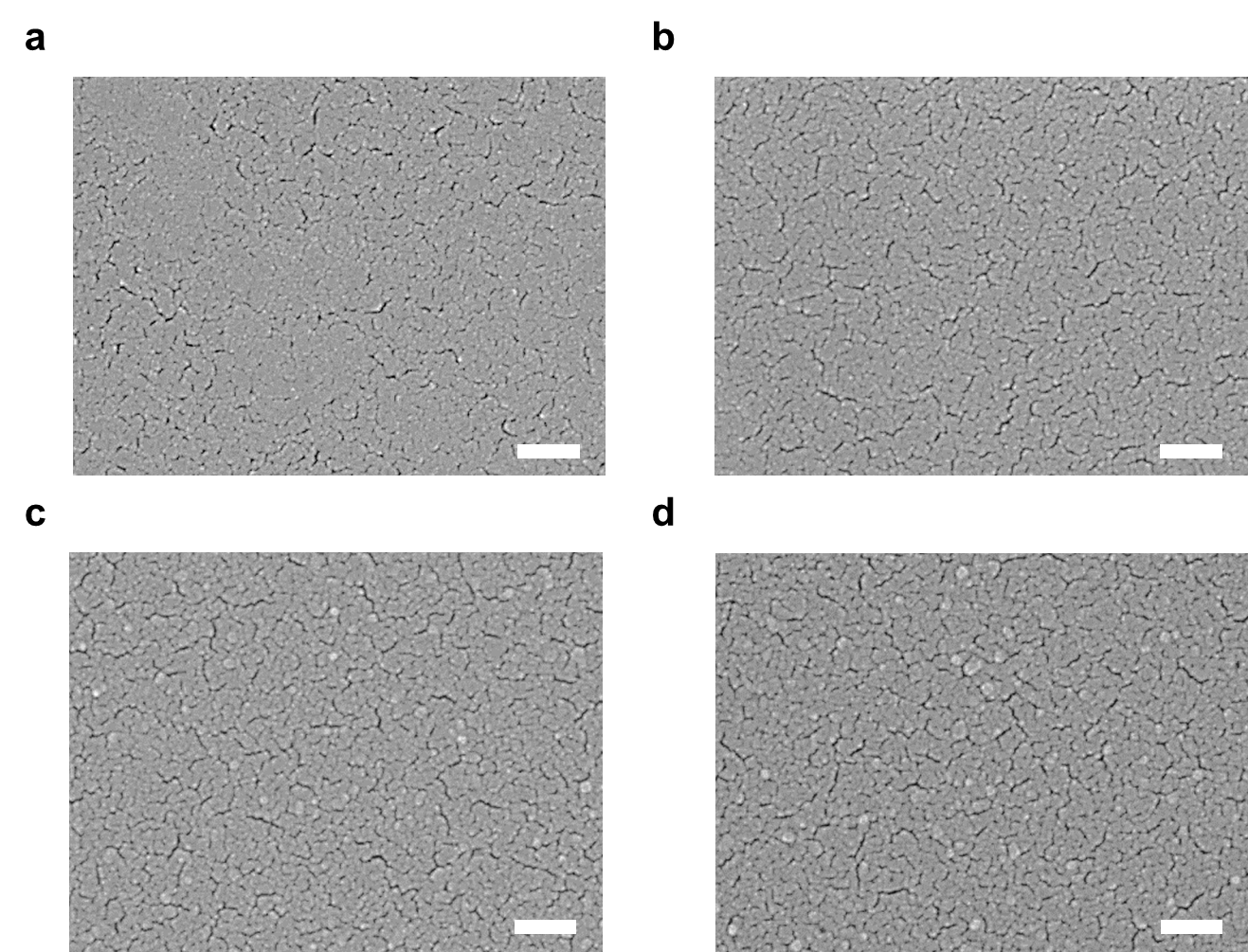


**Supplementary Fig. 5 | Effect of A-IDP process on nanostructure of quasi-2D perovskite thin films. a-d**, SEM image of perovskite thin films without (**a**) and with CCA-THF solution exposure time of 0 s (A-IDP) (**b**), 10 s (**c**), 20 s (**d**). Scale bar: 200 nm.


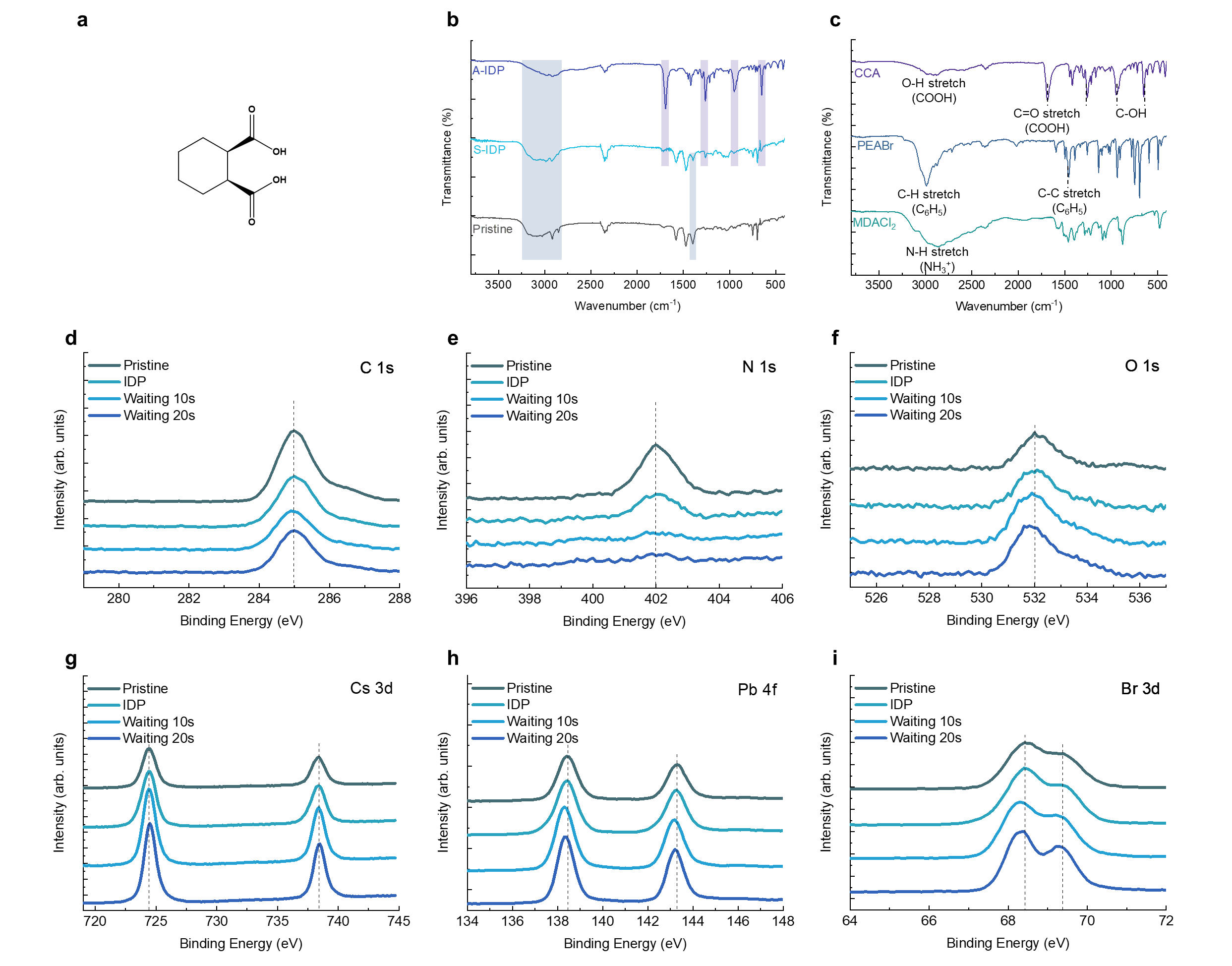


**Supplementary Fig. 6 | Chemical analysis on A-IDP process.
a**, Chemical structure of CCA. **b-c,** FT-IR spectra of perovskite films (**b**) and precursor powders (**c**). **d**, C 1s, e, N 1s, **f**, O 1s, **g**, Cs 3d, **h**, Pb 4f, and **i**, Br 3d XPS core-level spectra of quasi-2D perovskite thin films with different reaction condition of IDP process.

**
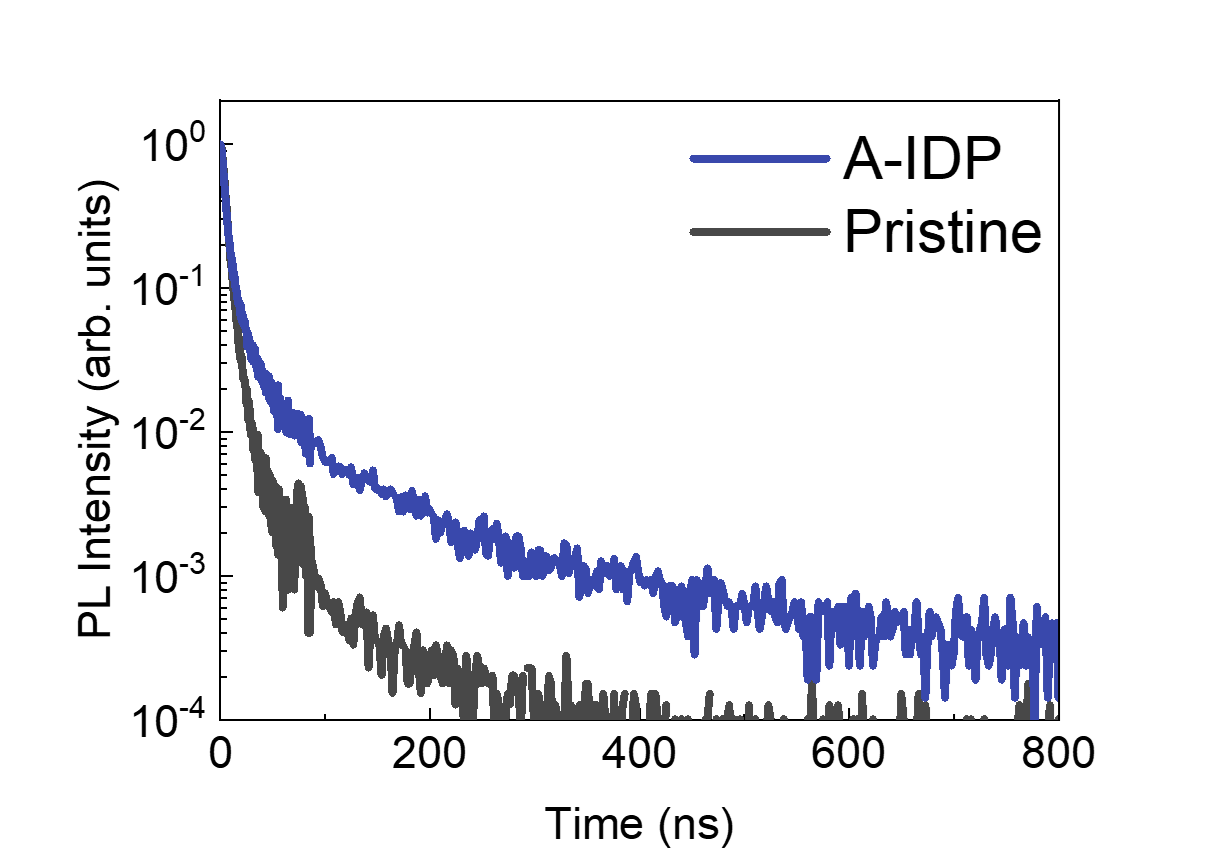
**

**Supplementary Fig. 7 | PL lifetime spectra of pristine and A-IDP films.** The increased PL lifetime in A-IDP films could be attributed to suppressed charge trapping by surface passivation from CCA molecules.


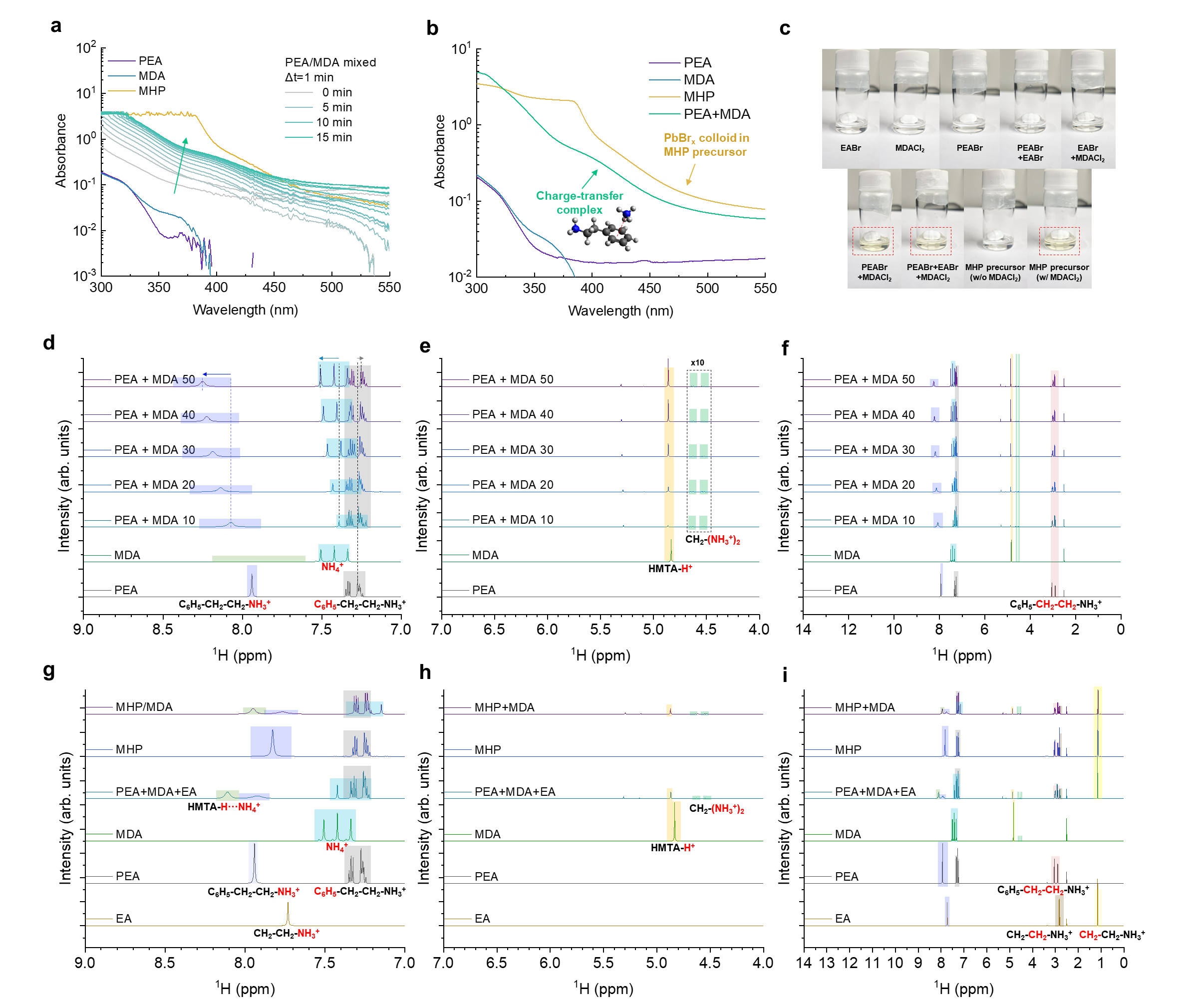


**Supplementary Fig. 8 | Solution-state chemical analysis on cation-π charge-transfer complex. a**, *In-situ* UV-Vis absorption spectra of PEA-MDA mixture solution and comparison with PEA, MDA, and metal halide perovskite (MHP) precursor solution. A new absorption peak that corresponds to MDA-PEA charge-transfer (CT) complex emerged. **b**, Steady-state UV-Vis absorption spectra of PEA-MDA mixture and precursor solution. **c**, Picture of mixture solutions used in MHP precursor solution. **d-f**, NMR spectra of PEA-MDA mixture solutions with different mixing ratios. **g-i**, NMR spectra of various combinations of mixture solution for perovskite precursor.


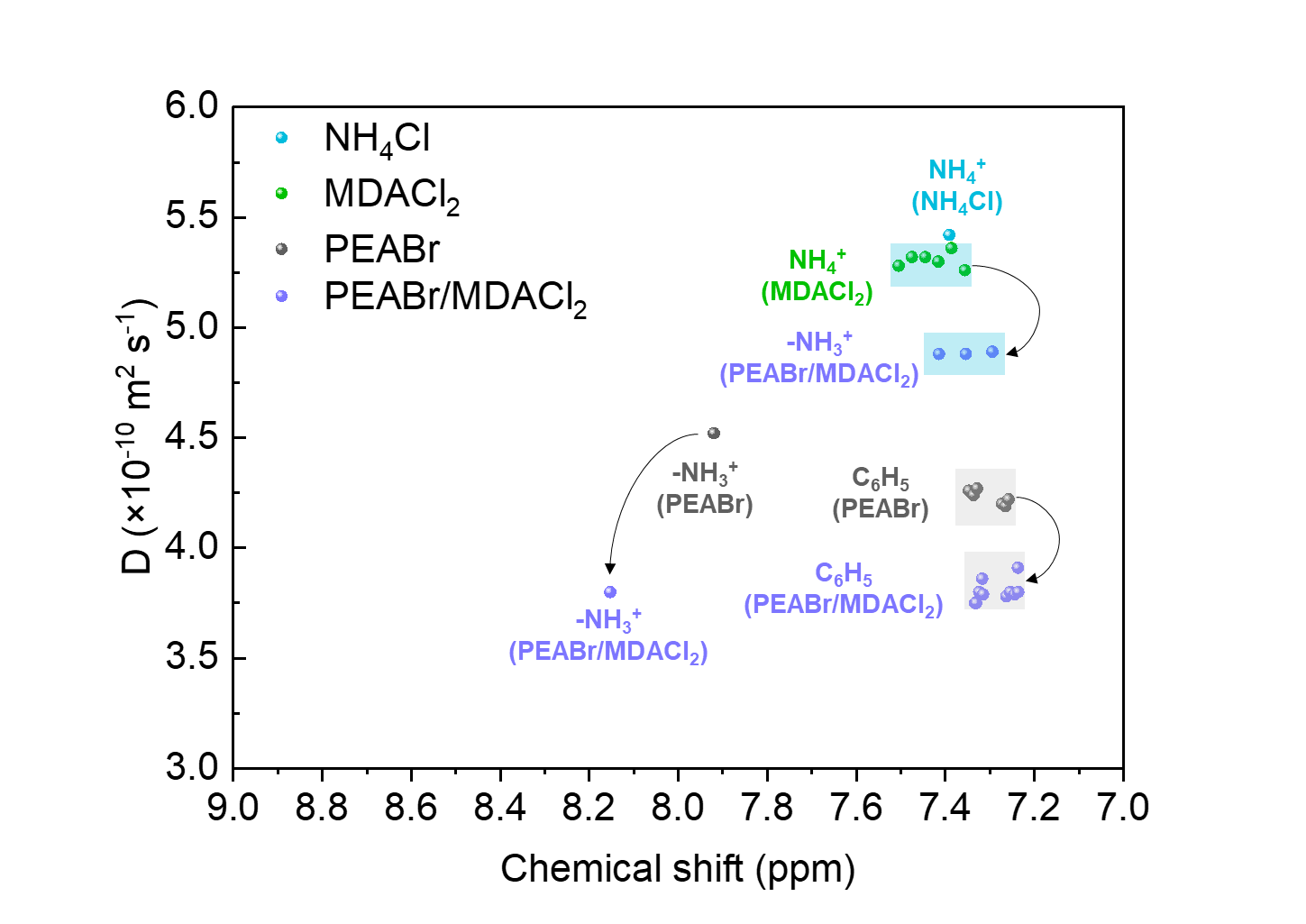


**Supplementary Fig. 9 | ^1^H 2D DOSY spectra of perovskite precursor solutions.** Each characteristic chemical shift was marked with each functional group of the precursor molecules. Overall diffusivity of precursor solutions was reduced after the addition of the MDACl_2_ additives.

**
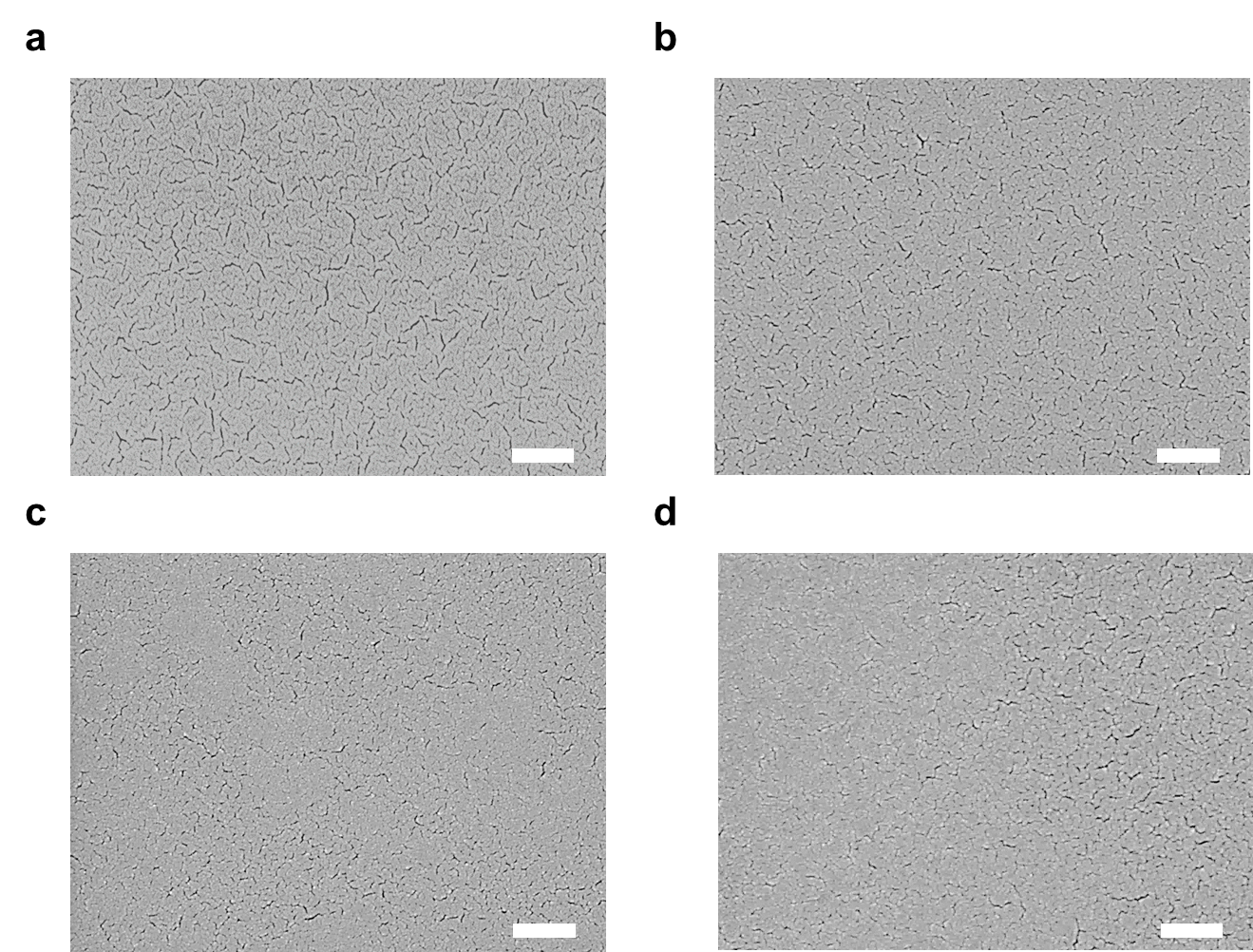
**

**Supplementary Fig. 10 | Effect of MDACl_2_ additives on nanostructure of perovskite thin films. a-d,** SEM image of perovskite thin films with **a**, 0 mol. %, **b**, 10 mol. %, **c**, 20 mol. %, **d**, 30 mol. % of MDACl_2_. Scale bar: 200 nm.


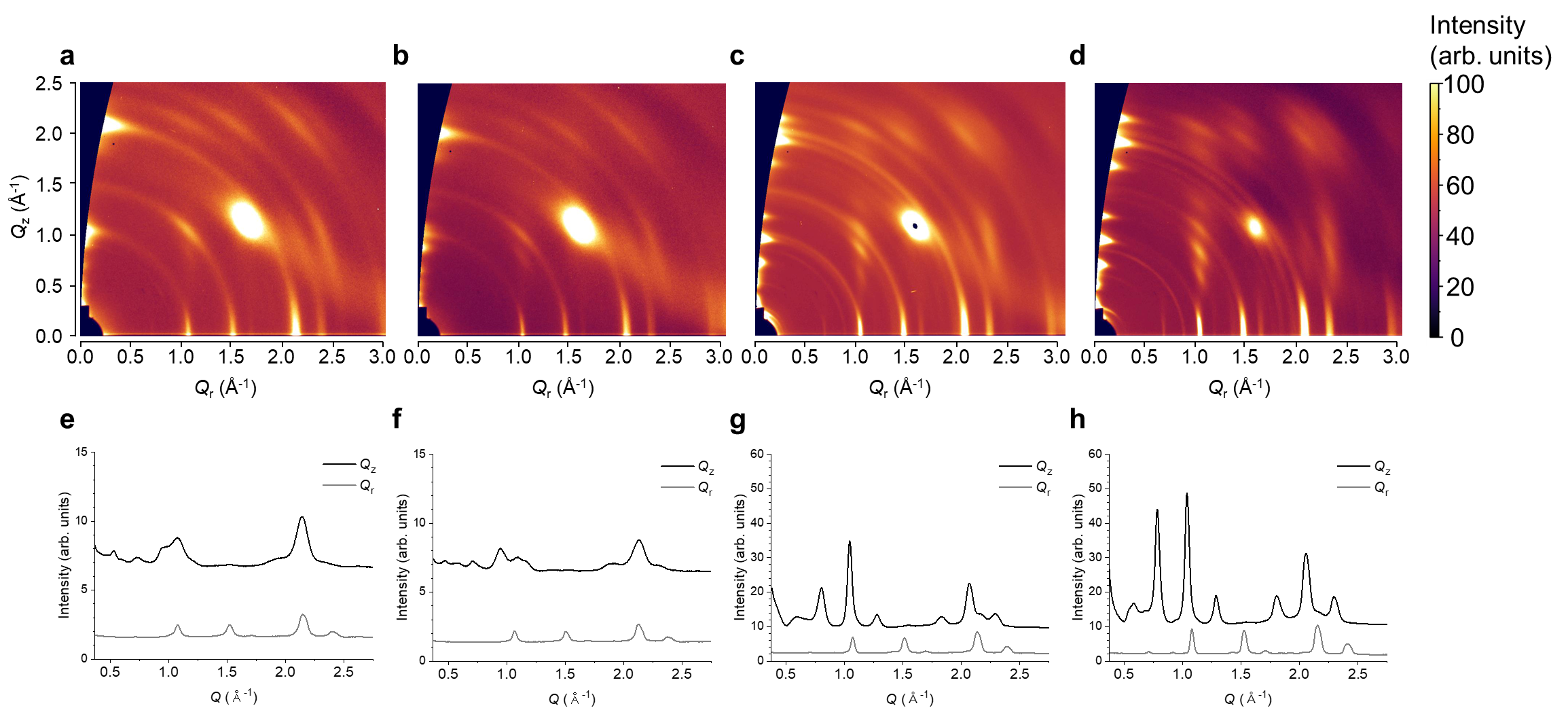


**Supplementary Fig. 11 | Effect of MDACl_2_ additives on crystal structure of perovskite thin films.**GIXRD pattern of perovskite thin films with **a**, 0 mol. %, **b**, 10 mol. %, **c**, 20 mol. %, **d**, 30 mol. % of MDACl_2_. 1D XRD pattern of perovskite thin films with **e**, 0 mol. %, **f**, 10 mol. %, **g**, 20 mol. %, and **h**, 30 mol. % of MDACl_2_.


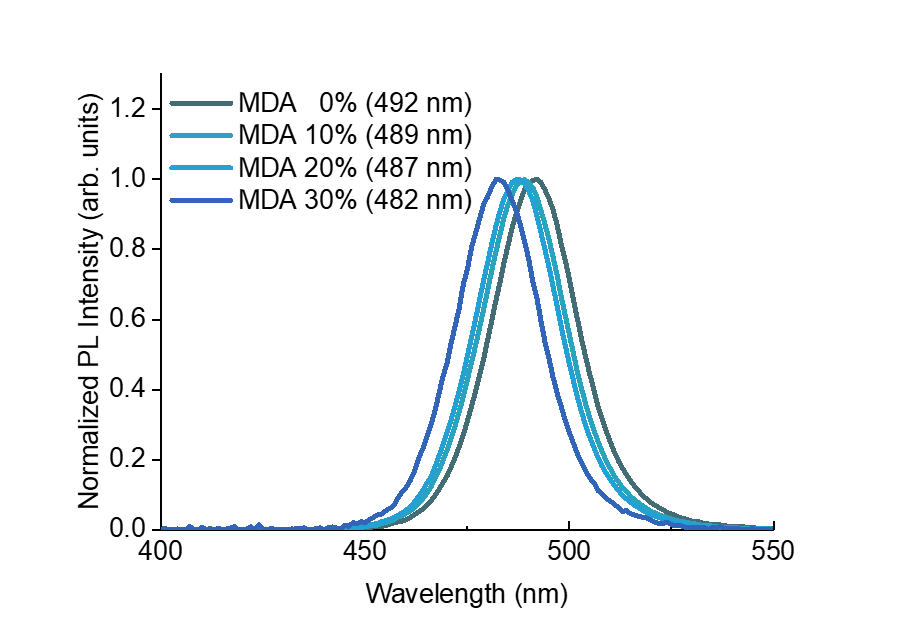


**Supplementary Fig. 12 | Steady-state PL spectra of pristine perovskite films with different amounts of MDACl_2_.** With increased amount of MDACl_2_ added, the PL spectra were gradually blue-shifted.


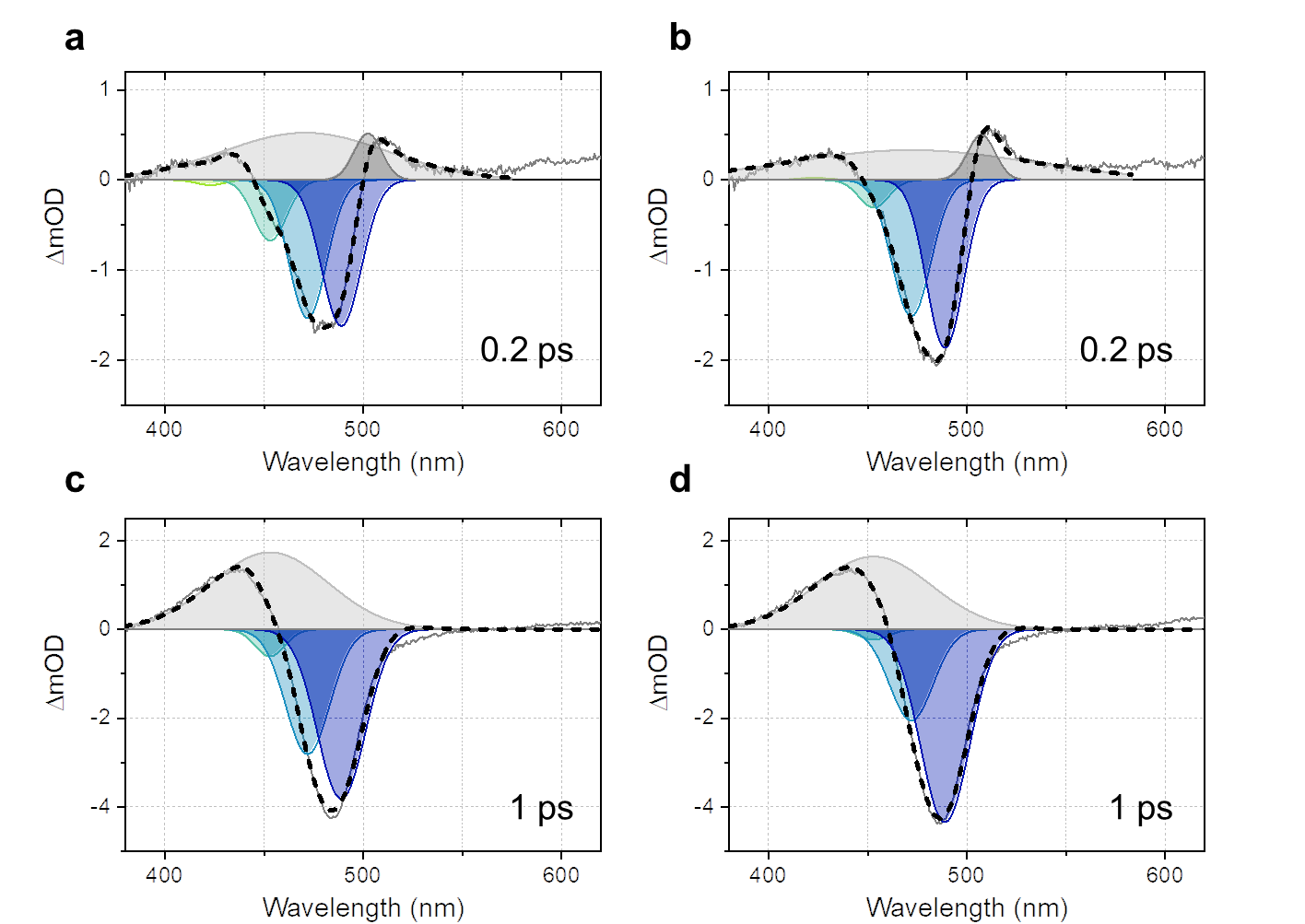


**Supplementary Fig. 13 | Effect of CCA on charge carrier dynamics of perovskite thin films.**Transient absorption spectra of S-IDP (**a, c**) and A-IDP (**b, d**) films at delay time 0.2 ps and 1 ps, fitted with multiple Gaussian functions. Average pump power was 0.9 mW, corresponding to an initial carrier density of 4.1 × 10^17^ cm^-2^. A distinct derivative-like feature appears at the red edge of the initial transient absorption spectra (<400 fs; **Extended Data Fig. 6a-b, Supplementary Fig. 8a-b**), providing insight into the magnitude of exciton-exciton interactions within the material.

Specifically, the exciton residing at the lowest energy state, induced by the probe pulse, engages in interactions with excitons pumped to higher energy states. This many-body interaction manifests as the carrier-induced Stark effect, resulting in a spectral transition shift^1^. The pronounced photoinduced absorption signal in the A-IDP film, compared to the pristine film, signifies an augmentation in the attractive Coulomb interaction between excitons, as evidenced by the red-shifted transition. This enhancement is particularly notable following the IDP treatment, especially between excitons occupying distinct energy levels. This enhancement aligns with the accelerated energy funnelling observed in the A-IDP film, as the Coulombic attraction between excitons in neighbouring crystals with varying phases (*n*) facilitates energy transfer through increased wavefunction overlap. In the broader context of device performance of light-emitting diodes, the efficient energy funnelling observed in the A-IDP film is elucidated as a Coulombically enhanced process. The chemically engineered organic layer diminishes the Van der Waals gap between quasi-2D crystals, enhancing accessibility to adjacent excitonic states.

While this could be misinterpreted as an elevated likelihood of Auger recombination (exciton-exciton annihilation), such an inference is not universally applicable. In the context of strongly confined colloidal quantum dots (QDs), where the biexciton Auger rate is known to be dependent on the QD volume, instances exist where the biexciton interaction energy exhibits size-independent, constant values^2^. This discrepancy is attributed to the involvement of poorly defined excitons generated in higher (continuum-like) energy states, disrupting the direct correlation between effective biexciton size and QD volume. Notably, the IDP treatment shifts the average phase distribution to higher *n*, relieving confinement across the crystal thickness and reducing the likelihood of Auger recombination.


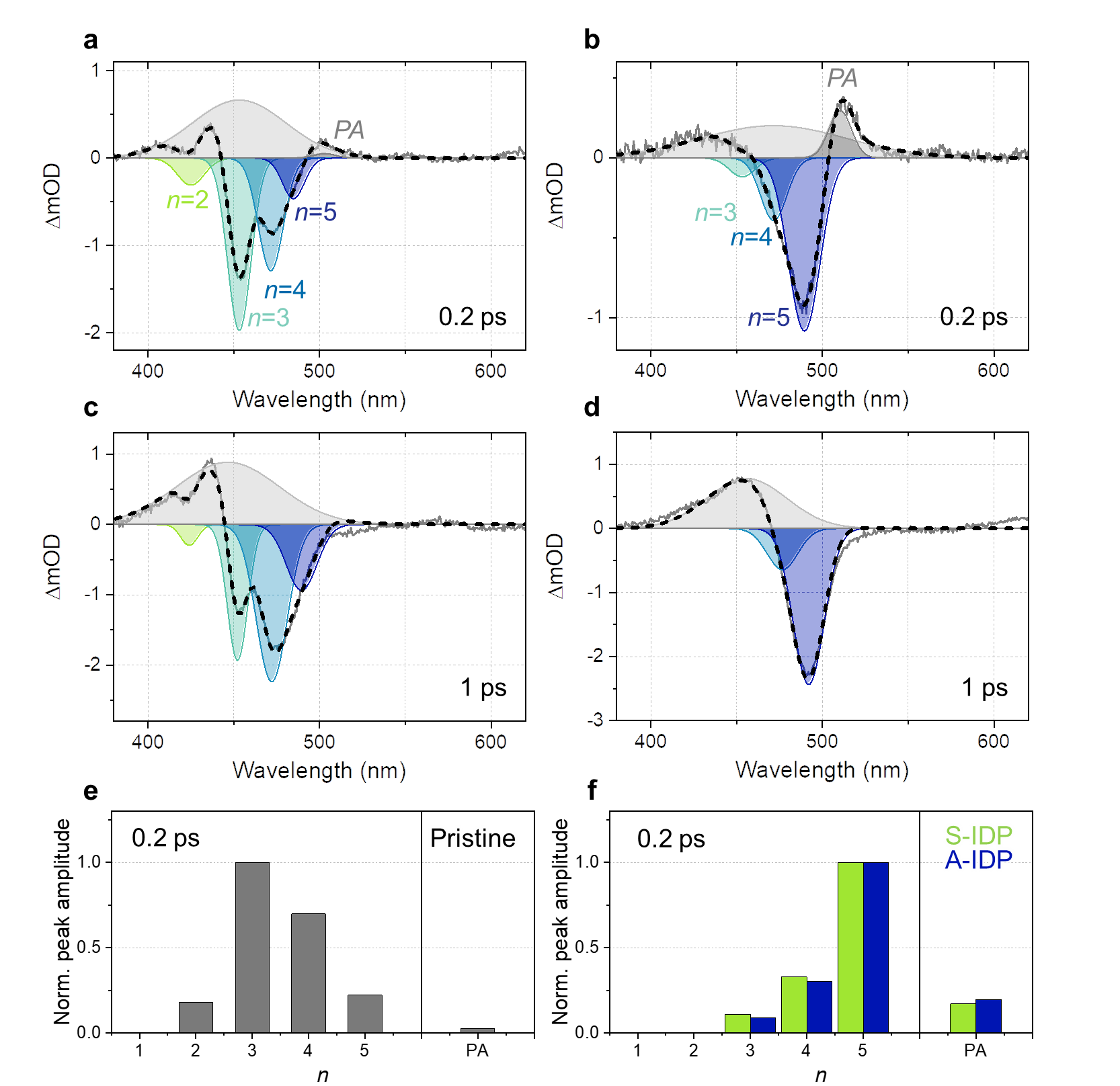


**Supplementary Fig. 14 | Phase distribution analysis with multiple Gaussian functions.
a-d**, Transient absorption spectra of pristine and A-IDP films at 0.2 ps (**a, b**) and 1 ps (**c, d**) fitted with multiple Gaussian functions. Average pump power was 0.32 mW, corresponding to the initial carrier density of 1.4 × 10^17^ cm^-2^. **e-f**, Relative peak amplitude of fitted Gaussian functions at each *n* phases normalized to the maximum. Photoinduced absorption (PA) feature at the low-energy side of the band gap, possibly arising from the attractive exciton-exciton (BX) interaction, is also shown with the relative peak amplitude.


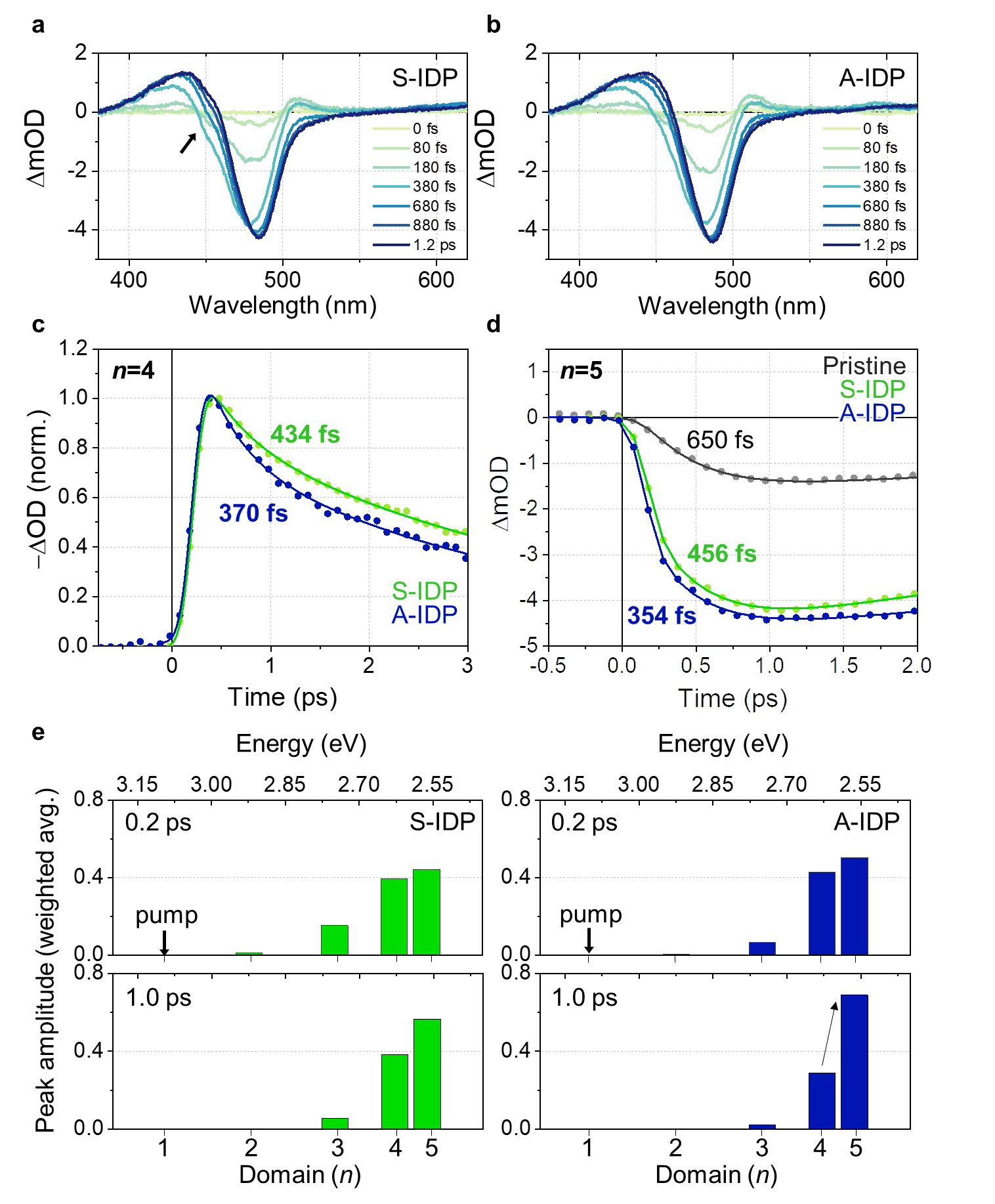


**Supplementary Fig. 15 | TA dynamics at higher pump fluences.**  **a-b**, Femtosecond TA spectra of S-IDP (**a**) and A-IDP (**b**) perovskite films. **c-d**, Normalized TA decay traces of pristine, S-IDP and A-IDP films probed at *n* = 4 (470 nm) (**c**) and at *n* = 5 (488 nm) (**d**). **e**, Carrier population distribution of S-IDP and A-IDP films presented by the weighted average peak area of TA spectra at delay times of 0.2 ps (upper panels) and 1 ps (lower panels).


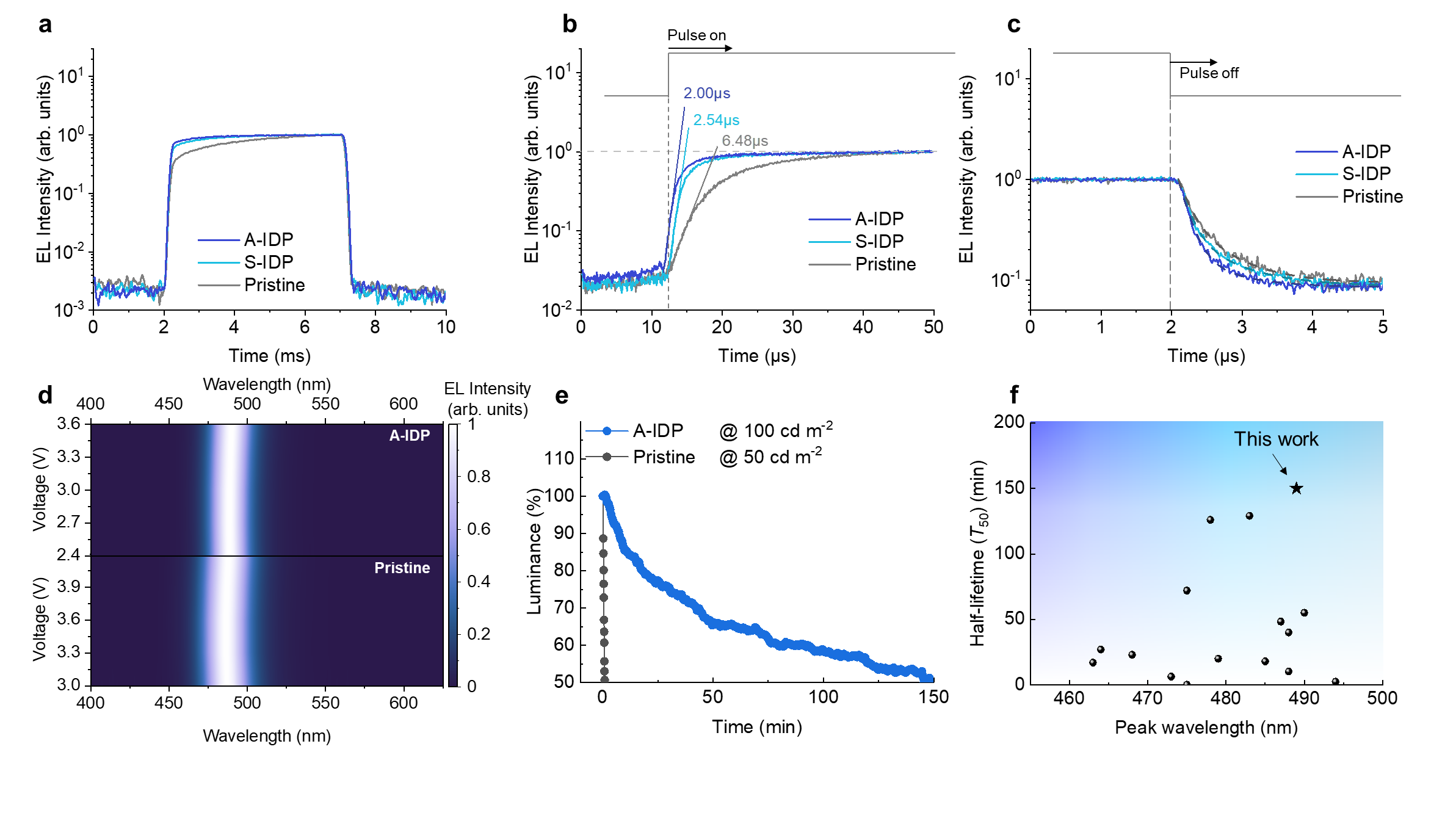


**Supplementary Fig. 16 | Electrical property of quasi-2D perovskite LEDs.
a-c**, Transient EL decay characteristics of quasi-2D PeLEDs. With homogeneous energy landscape and accelerated carrier transfer, S-IDP and A-IDP PeLEDs showed faster rising (fast charge injection) and falling (fast charge de-trapping) time of the turn-on and turn-off EL response. **d**, Normalized EL emission spectra versus voltage of quasi-2D PeLEDs. **e**, Lifetime versus time of quasi-2D PeLEDs. Inset: magnified view of lifetime curve at initial 5 min. **f**, Summary of the reported sky-blue to deep blue PeLEDs (peak emission wavelength < 495 nm) characteristics based on half-lifetime versus peak emission wavelength.


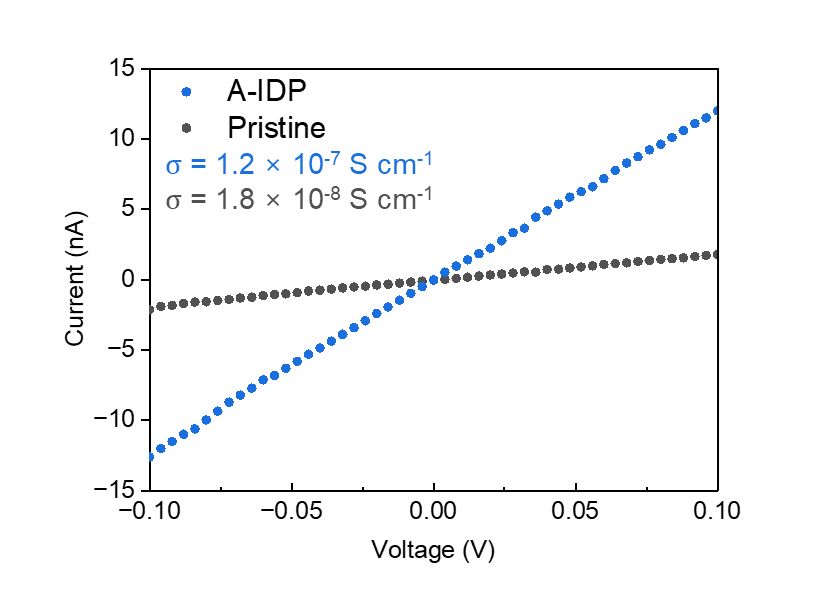


**Supplementary Fig. 17** | **Conductivity measurements for the lateral devices of quasi-2D perovskite thin films**. The lateral devices were fabricated by depositing two Au electrodes with 100 μm channel length on the perovskite films, where the conductivity was measured between two electrodes.


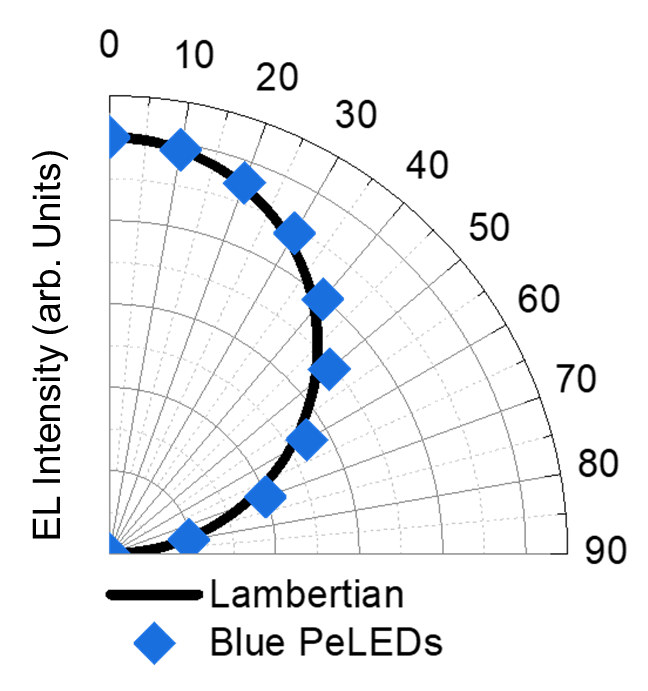


**Supplementary Fig. 18 | Angle-dependent EL intensity of blue PeLEDs.** The emission efficiency of the PeLEDs were calculated based on the difference between experimentally measured angle-dependent EL intensity profile and Lambertian profile.


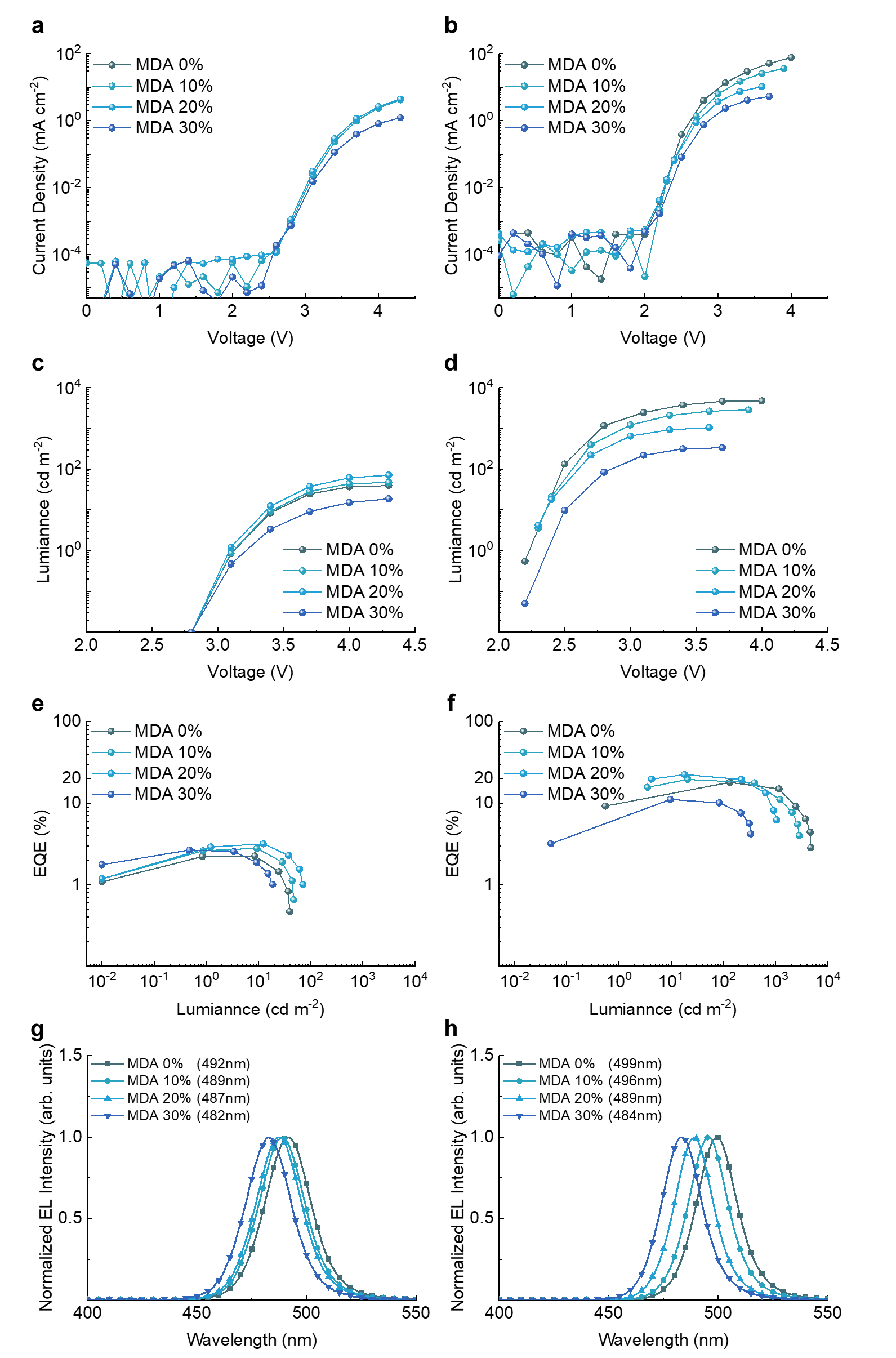


**Supplementary Fig. 19 | Device characteristics of PeLEDs with different amount of MDACl_2_. a-b,** Current density versus voltage, **c-d,** luminance versus voltage, **e-f**, EQE versus voltage, **g-h**, normalized EL spectra of pristine and A-IDP PeLEDs.


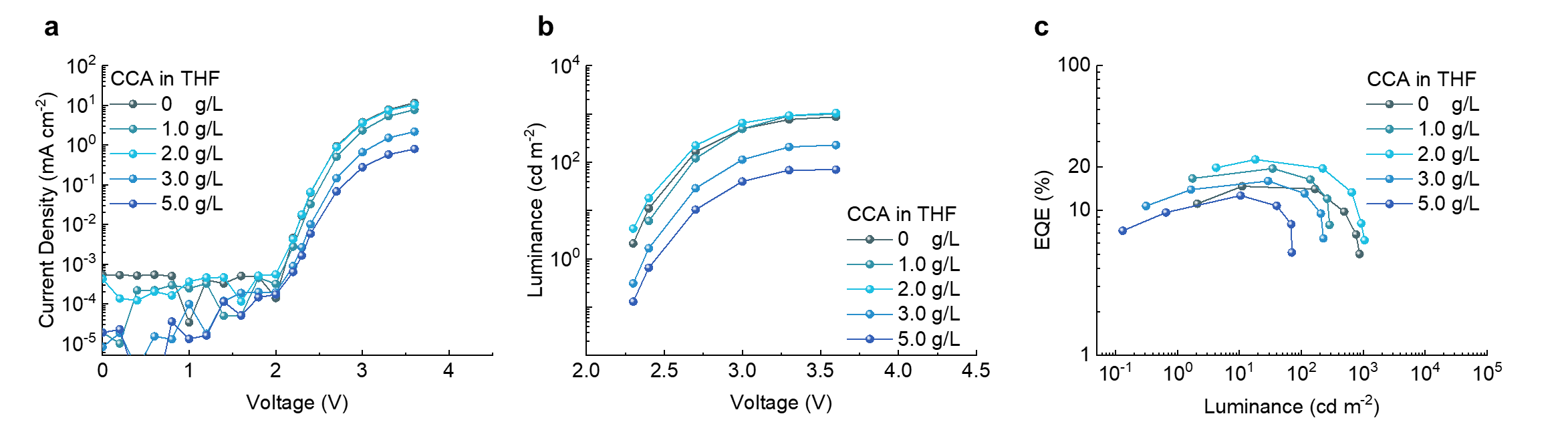
**Supplementary Fig. 20 | Device characteristics of PeLEDs with different amount of CCA. a,** Current density versus voltage, **b,** luminance versus voltage, **c**, EQE versus luminance of A-IDP PeLEDs with different amount of CCA in THF.


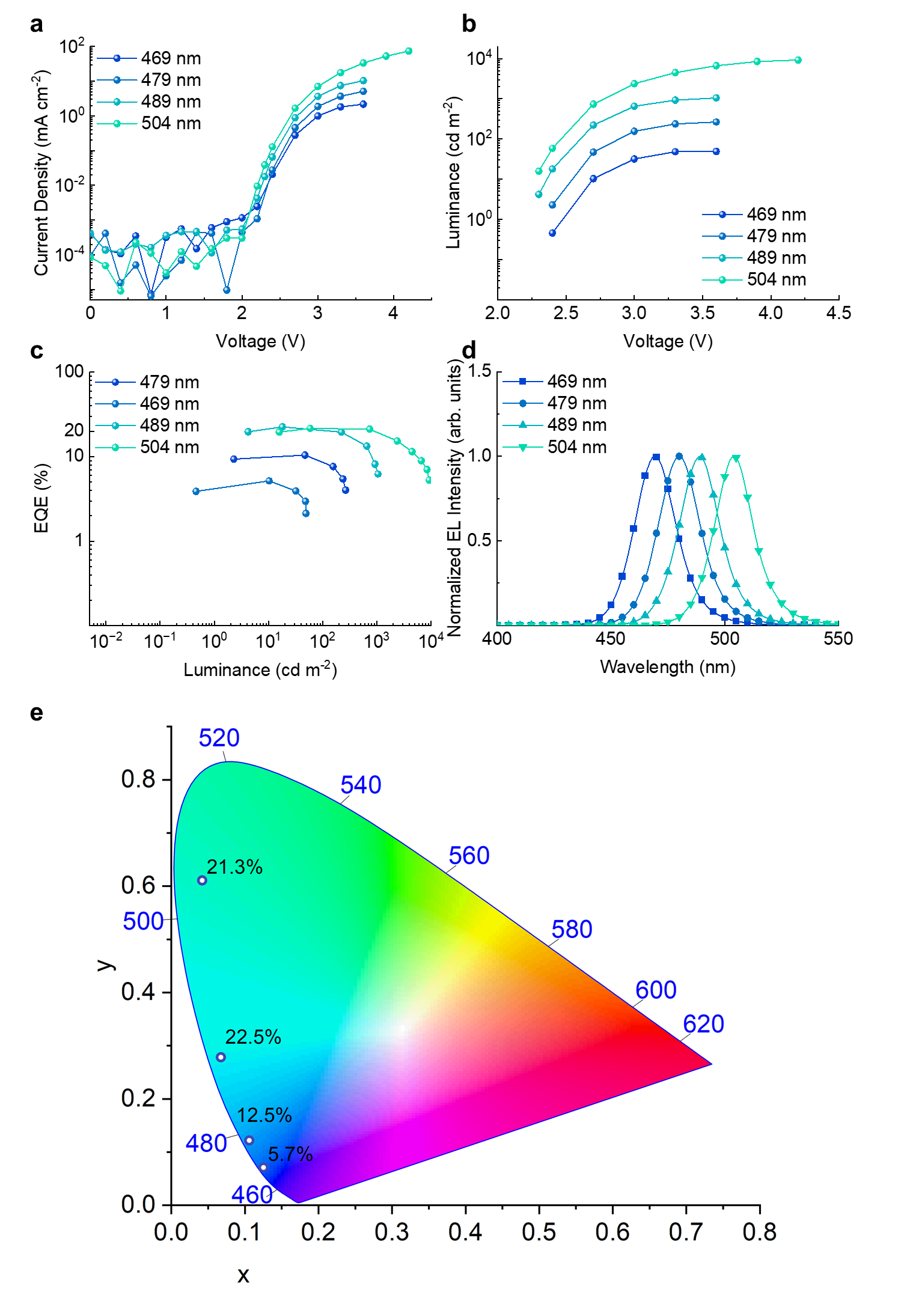


**Supplementary Fig. 21 | Emission wavelength tunability of A-IDP PeLEDs.**
**a**, Current density versus voltage, **b**, luminance versus voltage, **c**, EQE versus luminance, **d**, normalized EL spectra of A-IDP PeLEDs with emission peak centre at 504 nm. **e**, The CIE coordinates of A-IDP PeLEDs with emission spectra from green (504 nm) to deep-blue (469 nm) region. The green PeLEDs were realized by substituting CCA with naphthalene sulfonic acid (NSA) to facilitate formation of further high-*n* phases with smaller band gap, while the deep-blue and blue emitting PeLEDs were achieved by increased molar proportion of chlorides.

| Type of perovskites | *EQE*_max_ (%) | *L*_max_ (cd m^-2^) | *λ*_max_ (nm) | FWHM (nm) | EL linewidth (meV) |
| --- | --- | --- | --- | --- | --- |
| Pristine | 3.17 | 71.17 | 487 | 24.4 | 127.8 |
| S-IDP | 14.72 | 870.2 | 488 | 21.2 | 110.5 |
| A-IDP | 22.51 | 1051.3 | 489 | 20.8 | 108.0 |

**Supplementary Table 1 | Summarized electrical and luminance characteristics of PeLEDs.** *EQE*_max_: maximum EQE, *L*_max_: maximum luminance, *λ*_max_: Peak emission wavelength, FWHM: Full-width at half maximum of emission spectra.

**Supplementary Table 2 | Summarized electrical and luminance characteristics of pristine and A-IDP PeLEDs with different MDACl_2_ ratio.** *EQE*_max_: maximum EQE, *L*_max_: maximum luminance, *λ*_max_: Peak emission wavelength, FWHM: Full-width at half maximum of emission spectra.

| Perovskite | MDACl_2_  ratio | *EQE*_max_ (%) | *L*_max_ (cd m^-2^) | *λ*_max_ (nm) | FWHM (nm) |
| --- | --- | --- | --- | --- | --- |
| Pristine | 0% | 2.25 | 39.7 | 492 | 24.6 |
|  | 10% | 2.8 | 47 | 489 | 24.5 |
|  | 20% | 3.17 | 71.17 | 487 | 24.4 |
|  | 30% | 2.67 | 18.73 | 482 | 24.3 |
| A-IDP | 0% | 18.07 | 4,728 | 499 | 21.9 |
|  | 10% | 19.58 | 2847 | 496 | 21.3 |
|  | 20% | 22.51 | 1051 | 489 | 20.8 |
|  | 30% | 11.14 | 335 | 484 | 21.5 |

**Supplementary Table 3 | Summary of reported high-efficiency blue PeLEDs (without an outcoupling strategy).**

| Perovskite | Types | *λ*_max_ (nm) | FWHM  (meV) | *EQE*_max_ (%) | *T*_50_ | Ref. |
| --- | --- | --- | --- | --- | --- | --- |
| CsPbBr_3_ w/ X-MBA^+^  (X=F, Cl, Br, I) | QDs | 480 | 128.8 | 17.9 | 126 min @100 cd m^-2^ | 3 |
| CsPbBr_3_ w/ NABr | QDs | 479 | 115.7 | 12.3 | 20 min @ 90 cd m^-2^ | 4 |
| CsPbCl_1.5_Br_1.5_ w/ MBABr | QDs | 464 | 121.2 | 9.8 | 27 min @100 cd m^-2^ | 5 |
| CsPbBr_3_ as SAM active layer | NCs | 463 | 146.0 | 12 | 17 min @2 mA cm^-2^ | 6 |
| PEACs_x_EA_1-x_PbBr_3_ w/ CsCl | Quasi-2D | 486 | 125.4 | 16.07 | - | 7 |
| PEACs_x_EA_1−x_PbBr_3_ w/ GABA | Quasi-2D | 490 | 137.7 | 15.6 | 55.3 min @60 cd m^-2^ | 8 |
| CsPb(Br_0.6_Cl_0.4_)_3_ w/ MDAX (X=Cl, Br) | Quasi-2D | 475 | 136.7 | 14.2 | 72min @100 cd m^-2^ | 9 |
| PEACsPb(Cl/Br)_3_ w/ PEATFA | Quasi-2D | 468 | 117.5 | 11.87 | 23 min @1 mA cm^-2^ | 10 |
| PEACsPb(Cl/Br)_3_ | Quasi-2D | 494 | 132.3 | 15.5 | 154 s @100 cd m^-2^ | 11 |
| PEACs_1-x_Rb_x_Pb(Cl/Br)_3_ | Quasi-2D | 475 | 124.6 | 10.1 | ~100 s @100 cd m^-2^ | 12 |
| CsPb(Cl/Br)_3_ w/ PEABr-DPPABr | Quasi-2D | 473 | 125.6 | 8.8 | 6.3 min @100 cd m^-2^ | 13 |
| PEACsPb(Cl/Br)_3_ w/ PPT | Quasi-2D | 488 | 131.0 | 17.3 | - | 14 |
| PEACsPb(Cl/Br)_3_ w/ PFNBr | Quasi-2D | 485 | 124.7 | 11.2 | ~18 min @ 531 cd m^-2^ | 15 |
| PBACs_x_FA_1-x_PbBr_3_ | Quasi-2D | 483 | 147.2 | 9.5 | 250 s @ 100 cd m^-2^ | 16 |
| F-PEACsPb(Cl/Br)_3_ w/ PHDI | Quasi-2D | 487 | 132.4 | 14.82 | ~50 min @ 178 cd m^-2^ | 17 |
| PBACsPbBr_3_ w/ Ag NCs | Quasi-2D | 488 | 140.5 | 14.29 | 10.2 min @ 100 cd m^-2^ | 18 |
| PEACs_1−x_DA_x_PbBr_2.3_Cl_0.7_ | Quasi-2D | 488 | 128.0 | 14.71 | - | 19 |
| PEA_x_PA_2-x_CsPbBr_3_ w/ TBPO | Quasi-2D | 488 | 123.8 | 11.5 | ~40 min @ 100 cd m^-2^ | 20 |
| FPEACsPb(Cl/Br)_3_ w/ PPNCl | Quasi-2D | 483 | 140.5 | 21.4 | 129 min @ 100 cd m^-2^ | 21 |
| PEACs_x_EA_1-x_PbBr_3_ by A-IDP process | Quasi-2D | 489 | 108.5 | 22.5 | ~150 min @ 100 cd m^-2^ | This work |

**Supplementary References**

1. Klimov, V. I. Spectral and Dynamical Properties of Multiexcitons in Semiconductor Nanocrystals. *Annu. Rev. Phys. Chem.* **58**, 635–673 (2007).

2. Klimov, V., Hunsche, S. & Kurz, H. Biexciton effects in femtosecond nonlinear transmission of semiconductor quantum dots. *Phys. Rev. B* **50**, 8110–8113 (1994).

3. Jiang, Y. *et al.* Synthesis-on-substrate of quantum dot solids. *Nature* **612**, 679–684 (2022).

4. Dong, Y. *et al.* Bipolar-shell resurfacing for blue LEDs based on strongly confined perovskite quantum dots. *Nat. Nanotechnol.* **15**, 668–674 (2020).

5. Jiang, Y. *et al.* Unraveling Size‐Dependent Ion‐Migration for Stable Mixed‐Halide Perovskite Light‐Emitting Diodes. *Adv. Mater.* **35**, 2304094 (2023).

6. Wang, Y.-K. *et al.* Self-assembled monolayer–based blue perovskite LEDs. *Sci. Adv.* **9**, eadh2140 (2023).

7. Chu, Z. *et al.* Blue light-emitting diodes based on quasi-two-dimensional perovskite with efficient charge injection and optimized phase distribution via an alkali metal salt. *Nat. Electron.* **6**, 360–369 (2023).

8. Liu, S. *et al.* Zwitterions Narrow Distribution of Perovskite Quantum Wells for Blue Light‐Emitting Diodes with Efficiency Exceeding 15%. *Adv. Mater.* **35**, 2208078 (2023).

9. Zhang, L. *et al.* Manipulating Local Lattice Distortion for Spectrally Stable and Efficient Mixed‐halide Blue Perovskite LEDs. *Angew. Chemie Int. Ed.* **62**, e202302184 (2023).

10. Liu, Y. *et al.* A Multifunctional Additive Strategy Enables Efficient Pure‐Blue Perovskite Light‐Emitting Diodes. *Adv. Mater.* **35**, 2302161 (2023).

11. Peng, X. *et al.* Suppressed Energy Transfer Loss of Dion-Jacobson Perovskite Enabled by DMSO Vapor Treatment for Efficient Sky-Blue Light-Emitting Diodes. *ACS Energy Lett.* **8**, 339–346 (2023).

12. Yang, Y. *et al.* Highly Efficient Pure‐Blue Light‐Emitting Diodes Based on Rubidium and Chlorine Alloyed Metal Halide Perovskite. *Adv. Mater.* **33**, 2100783 (2021).

13. Wang, C. *et al.* Dimension control of in situ fabricated CsPbClBr_2_ nanocrystal films toward efficient blue light-emitting diodes. *Nat. Commun.* **11**, 6428 (2020).

14. Sun, S. *et al.* Highly Efficient Hybrid Perovskite/Organic Tandem White Light Emitting‐Diodes with External Quantum Efficiency Exceeding 20%. *Adv. Funct. Mater.* **33**, 2306549 (2023).

15. Yuan, S. *et al.* Efficient and Spectrally Stable Blue Perovskite Light‐Emitting Diodes Employing a Cationic π‐Conjugated Polymer. *Adv. Mater.* **33**, 2103640 (2021).

16. Liu, Y. *et al.* Efficient blue light-emitting diodes based on quantum-confined bromide perovskite nanostructures. *Nat. Photon.* **13**, 760–764 (2019).

17. Shen, Y. *et al.* Multifunctional Crystal Regulation Enables Efficient and Stable Sky‐Blue Perovskite Light‐Emitting Diodes. *Adv. Funct. Mater.* **32**, 2206574 (2022).

18. Zhang, F. *et al.* Engineering of Hole Transporting Interface by Incorporating the Atomic-Precision Ag_6_ Nanoclusters for High-Efficiency Blue Perovskite Light-Emitting Diodes. *Nano Lett.* **23**, 1582–1590 (2023).

19. Liu, B. *et al.* Lattice strain modulation toward efficient blue perovskite light-emitting diodes. *Sci. Adv.* **8**, eabq0138 (2022).

20. Ren, Z. *et al.* High-Performance Blue Quasi-2D Perovskite Light-Emitting Diodes via Balanced Carrier Confinement and Transfer. *Nano-Micro Lett.* **14**, 66 (2022).

21. Yuan, S. *et al.* Efficient blue electroluminescence from reduced-dimensional perovskites. *Nat. Photon.* **18**, 425–431 (2024).
